# Evaluation of the characteristics of workers injured on the job requiring hospitalization, and employer compliance with OSHA's reporting requirement for these work‐related hospitalizations

**DOI:** 10.1002/ajim.23447

**Published:** 2022-11-26

**Authors:** Mary Jo Reilly, Ling Wang, Kenneth D. Rosenman

**Affiliations:** ^1^ Department of Medicine, Division of Occupational and Environmental Medicine, College of Human Medicine Michigan State University East Lansing Michigan USA

**Keywords:** employer reporting, hospitalization, occupational injury and illness reporting, severe injury and illness

## Abstract

**Background:**

The Occupational Safety and Health Administration (OSHA) implemented a new standard in 2014 requiring employers to report nearly all work‐related inpatient hospitalizations within 24 h of the event. We examined the characteristics of the injured workers who were reported and the compliance of Michigan employers with the regulation.

**Methods:**

From 2016 to 2018, we compared reports of acute nonmotor‐vehicle work‐related injuries and illnesses from two independent datasets, employer reports to OSHA and the Michigan Multi‐Source Injury and Illness Surveillance System (MMSIISS) which collects injured worker hospital records from the 134 hospitals in Michigan. We matched records from employer reports to OSHA with the MMSIISS by employee's first and last name, company name, date of injury/illness, and type of injury/illness.

**Results:**

We identified 2887 workers hospitalized with severe injuries/illnesses from 2016 to 2018 in Michigan; 1260 workers were reported by employers to OSHA and 2238 workers were reported by hospitals to the MMSIISS. There was an overlap of 611 workers reported in both systems, while 649 workers were only reported by employers to OSHA and 1627 workers were only reported by hospitals to the MMSIISS. Employer compliance with the regulation over the 3 years showed a nonsignificant increase; from 42.0% to 43.6% to 45.0%. Fractures were the most frequent type of injury (1238, 42.9%), then head injuries, including skull fractures (470, 16.3%). The median length of hospital stay was 3 days. Manufacturing (709, 25.5%) and construction (563, 20.3%), accounted for the greatest number of hospitalizations. Employer‐reported cases to OSHA significantly undercounted hospitalized workers in agriculture, forestry, fishing, and hunting; construction; finance and insurance; real estate and rental and leasing; administrative and support and waste management and remediation services; arts, entertainment, and recreation; accommodation and food services; and other services except public administration. Companies with 250 or more employees were significantly more likely to comply and small companies with 10 or fewer employees were significantly less likely to comply with the reporting rule. Enforcement inspections at 465 of the workplaces where a hospitalization had occurred resulted in $1,017,835 in fines and identified 608 violations. Of the 465 inspections, 246 (52.9%) of the employers had not corrected the hazard before the inspection.

**Conclusions:**

This study identified that workers sustained severe injuries and illnesses on the job and that over half of the companies where a worker suffered an injury/illness leading to hospitalization were not in compliance with OSHA's reporting regulation. Furthermore, at the time of an inspection 1–5 months later, 50% of the companies had not corrected the hazard causing the hospitalization. Improvement in the reporting of work‐related injuries/illnesses that result in hospitalization will identify more ongoing hazards in the workplace and improve where to focus preventive actions.

## 1 BACKGROUND

Work‐related injuries and illnesses are preventable. Workers who sustain a severe injury or illness resulting in hospitalization are indicators of hazardous work conditions. These hospitalizations place a burden on the workers, their families, and their workplaces. Identifying work‐related hospitalizations can help better understand what workplace corrections in safety and health are needed to prevent other workers from sustaining similar injuries and illnesses.

In 2014, the federal Occupational Safety and Health Administration (OSHA) implemented a new reporting requirement for acute severe injuries and illnesses that resulted in inpatient hospitalization. All OSHA state plans were required to adopt the new requirement. In 2015 Michigan OSHA (MIOSHA), a state‐plan state, implemented the new employer reporting requirement. This is the first evaluation of employer compliance with the reporting requirement for hospitalizations, either at the federal or state‐plan level.

Studies have found that employers vary greatly in their compliance with occupational injury and illness regulations involving reporting and recordkeeping.^1^, ^2^ In the state of Washington, researchers found that 90% of employers failed to correctly record work‐related injuries in the Bureau of Labor Statistics Survey of Injuries and Illnesses (BLS SOII).^3^ In a survey of four states, researchers found that less than half of employers that were required to maintain an injury and illness log did so.^4^ Reasons for incorrect or incomplete reporting included business practices that encourage reporting low rates of injury and illness, poor organization of records, and poor understanding of reporting requirements. From 2009 to 2012, federal OSHA conducted a National Emphasis Program for Recordkeeping and found that approximately half of the employers had recordkeeping violations.^5^ Reasons for poor recordkeeping included employees not reporting injuries due to fear of repercussions and employer medical management policies, where an in‐house medical department existed. Poor recordkeeping and reporting adversely affect workplace safety and health.

This manuscript examines the characteristics of workers who experience a severe injury or illness resulting in hospitalization. It compares hospitalizations submitted directly by employers to the state OSHA plan in compliance with the new reporting requirement to hospital‐reported cases, which are regularly submitted to the state as part of Michigan's Multi‐Source Injury and Illness Surveillance System (MMSIISS). Finally, we examine the outcomes when an OSHA enforcement inspection is conducted at the workplaces of injured workers.

## 2 METHODS

Federal OSHA adopted a nationwide employer reporting regulation in 2014; on September 1, 2015, MIOSHA as required by federal OSHA adopted similar regulations that require employers to report any employee who has an inpatient (overnight) hospitalization within 24 h of their acute work‐related injury or illness to a special hotline or online portal. Motor vehicle‐related work injuries are excluded. Employers under the jurisdiction of Michigan OSHA include all public and private sector places of employment in the state of Michigan even if the employer's corporate office is located out of state, except for federal employees, employees of the United States Postal Service, domestic employees, maritime workers, and mine workers, which are all subject to federal OSHA jurisdiction. Tribal businesses and the self‐employed who are the sole employee of their company are not subject to state or federal OSHA. The authority to require employer reporting was based on MIOSHA Administrative Standard Part 11, /R 408.22101 et seq., Recording and Reporting of Occupational Injuries and Illnesses. Before this new reporting requirement, employers were required to report inpatient hospitalizations of three or more employees associated with a single work‐related incident, to OSHA within 8 h. Employers were made aware of the new reporting requirement through extensive outreach via the MIOSHA website, a press release, emails to employers, through outreach from the MIOSHA Consultation, Education and Training Division, and through other educational venues such as the annual Michigan Safety Conference, which hosts thousands of health and safety professionals each year.

Separate from this employer‐reporting requirement, Michigan has maintained a surveillance system, the MMSIISS, for work‐related conditions, based on laws adopted in 1978 for illnesses and in 2010 for traumatic injuries. The authority to identify and collect the hospital reports is based on the 1978 Michigan Public Health Code (Article 368, Part 56, P.A. 1978) and the 2010 Michigan Public Health Code (Article 369, R325.301‐306, P.A. 1978). All 134 Michigan hospitals report work‐related hospitalizations to the MMSIISS on a quarterly basis. Hospitalized cases could be employed under any jurisdiction, including state and federal OSHA as well as tribal establishments and the self‐employed. Case identification from hospitals primarily relied on the identification of workers' compensation as the expected payer and a discharge diagnosis of an acute injury/illness with an overnight stay. For three work‐related injuries, burns, skull fractures, and crushing injuries, the MMSIISS program reviewed the medical records for any injured inpatient ≥14 years regardless of payer source. To align with employer reporting to OSHA, cases reported by hospitals were excluded if the individual was self‐employed, worked for an employer not covered by Michigan OSHA, the hospitalization did not occur within 24 h of the injury, or the injury was the result of a motor vehicle crash. The first hospitalization record was abstracted for each worker. Most workers were not transferred to another hospital. However, if a worker was transferred to another hospital for a higher level of care, the hospital record where the worker was transferred was used. Each worker was only counted once.

Both employer reports to OSHA and the MMSIISS reporting systems used the same definition of an acute work‐related injury or illness. Both systems collected the date of injury, the injured employee's name, the company name, and a description of the injury type and body part. Hospitals collected additional data that were not collected in the OSHA system: age, gender; race; Hispanic ethnicity; and and date of admission and date of discharge. OSHA also does not collect the name of the hospital to which the worker was admitted for care. Even though the OSHA employer database does not include gender, gender was largely imputed based on the injured workers' first names. Individuals whose workplace could not be identified in the MMSIISS medical records were contacted to obtain employer information. Not all workplaces could be identified in MMSIISS despite outreach to injured workers. The length of stay (LOS) for workers reported by hospitals to the MMSIISS was calculated based on the date of admission to the date of discharge. A North American Industry Classification System (NAICS) code was assigned to each employer. For employers that had been inspected, we used the NAICS code listed in the OSHA inspection report. For employers that were not inspected, a variety of resources were used to identify the NAICS code, including online lookups in the OSHA Integrated Management Information System (IMIS) where a company may have been inspected in the past unrelated to the reporting requirement, manta.com and prior information housed at the MMSIISS surveillance center on industries associated with other work‐related conditions in Michigan. Company size was coded as small (1–10 employees), medium (11–249 employees), and large (250+ employees). Company size was identified through manta.com and prior information housed at the MMSIISS surveillance center on industries associated with other work‐related conditions in Michigan. The data for the hospitalized workers reported to the MMSIISS was abstracted from the medical record and entered into an ACCESS database. An EXCEL file of the data reported by employers to OSHA was obtained from OSHA and uploaded into an ACCESS database. *χ* ^2^ tests were used to test the differences in compliance rates of employers reporting to OSHA by age, gender, race, and Hispanic ethnicity for the 2238 cases reported by hospitals to the MMSIISS. Logistic regression was employed to obtain predicted 95% confidence intervals (CIs) of compliance rates of employer reporting to OSHA by injury type, industry, and company size, separately using SAS version 9.4, for the 2887 workers reported either through employers to OSHA or by hospitals to the MMSIISS. Nonoverlapping 95% CIs were considered statistically significant at *α* = 0.05 level. For the 2238 hospitalized workers reported by hospitals to the MMSIISS, the Mann–Whitney *U* test was used to compare the distribution of LOS between the employer reports to OSHA and those just reported to the MMSIISS but not reported by employers to OSHA.

Using the first 3 full years of data, 2016 to 2018, we matched the hospitalized injuries and illnesses reported by the employer to OSHA to the hospitalized injuries and illnesses reported to the MMSIISS, by employee first and last name, company name, injury date, and type of injury. Allowance was made for slight differences in the elements used to match the workers between the two systems. For example, one reporting system might list a temporary employment agency as the employer while the other might list the host employer. In some cases, the company's parent name was listed in one system while it was listed under its DBA name (doing business as). In other cases, the hospital did not have the company name but given the other details (patient name, date of injury, and description of injury), it was clearly a match across the two systems. Similarly, in very few instances first and last names were transposed in the employer reports to OSHA, and there were a few slight name misspellings. For all injured workers, though, the match was clearly a true match. The demographics, injury/illness and industry characteristics of cases reported by employers to OSHA were compared to cases reported to the MMSIISS by hospitals.

Onsite OSHA inspections were conducted at some of the companies where workers sustained an injury/illness requiring hospitalization. OSHA inspections can be initiated for a variety of reasons including but not limited to an employee complaint; an employer report to OSHA; a referral from a physician or health and safety professional; as a follow‐up to a previous inspection; because the company is in a high‐risk industry group; because the company falls under a special emphasis program; or the company is on a list for a planned inspection that year. MIOSHA performed enforcement inspections at companies associated with injured workers from both the MMSIISS hospitalization reports and the employer reports to OSHA. Inspections based on employer reports to OSHA were conducted closer to the time of injury, typically within one month, since the law requires employer reporting within 24 h of the injury/illness. Inspections based on hospital reports to the MMSIISS were conducted within 6 months of the hospitalization, given that hospitals submitted their reports on a quarterly basis. Inspections were only conducted where the injury appeared to be caused by a correctable hazard. The IMIS was used to look up each company that was inspected. Inspection reports contained information on the company name, type of company (NAICS code), specific violations identified and whether they were serious, total fines assessed, and, in some cases, contained a narrative of the events surrounding the injury. Inspection data from IMIS were abstracted and entered into an ACCESS database.

This public health activity was considered exempt by the Michigan State University Human Subjects Board.

## 3 RESULTS

**Figure 1:**
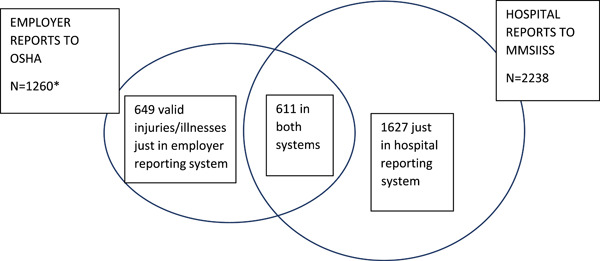
Overlap of two reporting systems for 2887 severe work‐related injuries and illnesses: Employer reports to OSHA and hospital reports to the Michigan Multi‐Source Injury and Illness Surveillance System (MMSIISS), Michigan 2016–2018. *An additional 257 injuries/illnesses reported by employers to OSHA were not valid (e.g., motor vehicle crashes, emergency department visits only). OSHA, Occupational Safety and Health Administration. [Color figure can be viewed at wileyonlinelibrary.com]

There were 2887 workers hospitalized with severe injuries/illnesses from 2016 to 2018 in Michigan; 1260 workers were reported by employers to OSHA and 2238 workers were reported by hospitals to the MMSIISS. There was an overlap of 611 workers reported by both employers and the hospitals, while 649 workers were only reported by employers to OSHA and 1627 workers were only reported by hospitals to the MMSIISS. Figure 1 shows the overlap of the 2887 severe work‐related injury and illness hospitalizations reported by employers and hospitals. Employers reported an additional 257 workers, who were excluded because a review of these reports showed that some employers reported any type of hospitalization; there were 184 reports of heart attacks, nonacute injuries, or injuries from motor vehicle collisions, and some employers reported all hospital encounters such as emergency department visits or observation even when there was not an overnight stay; for 73 the description of the injury severity suggested that the injury did not necessitate hospitalization or the hospitalization occurred more than 24 h after the injury. Overall, in 2016, there were 880 hospitalized workers with 370 (42.0%) reported by employers to OSHA, 932 with 406 (43.6%) reported by employers to OSHA in 2017, and 1075 with 484 (45.0%) reported by employers to OSHA in 2018. Compliance with employer reporting of injuries to OSHA did not significantly increase over the 3 years.

Tables 1 and 3 compare the subset of 611 reports from employers to OSHA that matched an injured worker in the 2238 hospitalizations in MMSIISS for demographics (age, gender, race, and Hispanic ethnicity [Table 1]) and LOS (Table 3), which is not available in the employer reports to OSHA but is only available in the MMSIISS. Where information is available for hospitalized workers regardless of which system in which they were reported, the results compare employer compliance of reporting to OSHA with the total number of 2887 hospitalizations from 2016 to 2018 for injury type (Table 2), industry type (Table 4), and company size (Table 5).

**Table 1:** Demographics of 2238 work‐related hospitalizations in the Michigan Multi‐Source Injury and Illness Surveillance System (MMSIISS) by employer reporting status to OSHA, 2016–2018

	MMSIISS (*n* = 2238)						
	Did the employer report to OSHA?						
	Yes (*n* = 611)		No (*n* = 1627)			All (*n* = 2238)	
Gender	Average age, SD (years)	No. (%)	Average age, SD (years)		No. (%)	Average age, SD (years)	No. (%)
Male	43.9, ±14.6	515 (84.3)	44.6, ±15.5		1299 (79.8)	44.4, ±15.2	1814 (81.1)
Female	50.0, ±13.9	96 (15.7)	51.0, ±16.3		328 (20.2)	50.8, ±15.8	424 (18.9)
Total	44.8, ±14.7	611	45.9, +15.8		1,627	45.6, ±15.5	2238
Race							
White		268 (80.2)			850 (83.7)		1118 (82.9)
Black		52 (15.6)*			120 (11.8)		172 (12.8)
Asian		4 (1.2)			13 (1.3)		17 (1.3)
Other		10 (3.0)			32 (3.2)		42 (3.1)
Total		334			1015		1349
Unknown race		277			612		889
Hispanic ethnicity							
Yes		16 (9.5)**			65 (11.7)		81 (11.2)
No		152 (90.5)			491 (88.3)		643 (88.8)
Total		168			556		724
Unknown ethnicity		443			1071		1514

Abbreviation: OSHA, Occupational Safety and Health Administration.

* *p* = 0.007

** *p* = 0.008.

**Table 2:** Injury type of 2887 work‐related hospitalizations by reporting source, Michigan 2016–2018

	Reporting source				
	MMSIISS only (*n* = 1627)	MMSIISS and employer reports to OSHA (*n* = 611)	Employer reports to OSHA only (*n* = 649)	All (*n* = 2887)	% Employers in compliance with reporting rule
Injury/illness type	No. (%)	No. (%)	No. (%)	No. (%)	% (95 CI)
Fracture	704 (43.3)	263 (43.0)	271 (41.8)	1238 (42.9)	43.1 (40.4–45.9)
Head injury and skull fracture	300 (18.4)	100 (16.4)	70 (10.8)	470 (16.3)	▼36.2 (31.8–40.5)
Crushing	145 (8.9)	89 (14.6)	41 (6.3)	275 (9.5)	47.3 (41.4–53.2)
Burns	122 (7.5)	54 (8.8)	52 (8.0)	228 (7.9)	46.5 (40–53)
All other	356 (21.9)	105 (17.2)	215 (33.1)	676 (23.4)	47.3 (43.6–51.1)
Totals and overall compliance	1627	611	649	2887	43.6 (41.8–45.4)

*Note*: ▼ indicates significantly lower compliance rate than overall compliance rate.

Abbreviation: OSHA, Occupational Safety and Health Administration.

The injured workers ranged from 15 to 92 years of age. The average age of the 2238 hospitalized workers reported to the MMSIISS and the 611 that were also reported by employers to OSHA were similar; in the MMSIISS, the overall average age was 44.4 years for males and 50.8 for females (Table 1). For those reported by employers to OSHA, the average age was 43.9 years for males and 50.0 for females. There was no statistical difference in employer compliance to report by the age of the injured worker. Reported hospitalizations more often occurred among men than women, 81.1% versus 18.9%; there was no statistical difference in employer compliance with reporting by the gender of the injured worker. The race was unknown for 889 (39.7%) of the 2238 workers in the MMSIISS and was unknown for 277 (45.3%) of the 611 cases reported by employers to OSHA. Hispanic ethnicity was unknown for 1514 (67.6%) of the 2238 workers in the MMSIISS and was unknown for 443 (72.5%) of the 611 cases reported by employers to OSHA. For reports where race and Hispanic ethnicity were known, the percentage of Black workers was significantly larger (15.6% vs. 11.8%) and the percentage of those of Hispanic ethnicity was significantly smaller (9.5% vs. 11.7%) among workers reported by employers to OSHA compared to reports of workers in the MMSIISS (Table 1).

Hospitalized workers sustained a variety of severe injuries/illnesses. Fractures accounted for the greatest percentage of injured workers, with 1238 (42.9%), followed by head injuries including skull fractures with 470 (16.3%), crushing injuries with 275 (9.5%), burns with 228 (7.9%), and all other injuries accounting for 676 (23.4%) of the 2887 hospitalizations (Table 2). A total of 1260 (611 plus 649) workers reported by their employer in compliance with the OSHA regulation had a similar distribution of type of injury, except for head injuries including skull fractures, which had a significantly lower compliance rate of employer reporting to OSHA (Table 2). The percentage of employers in compliance with the reporting rule varied from a low of 36.2% among head injuries including skull fractures to a high of 47.3% each among crushing and all other injuries.

The median LOS for the 2238 workers in the MMSIISS was 3 days, ranging from a low of 2 days among workers with head injuries including skull fractures to 3 days for all other types of injuries (Table 3). The total number of days hospitalized for the 2238 workers in the MMSIISS was 10,435 days. Within the subset of 611 workers in the MMSIISS that were also reported by employers to OSHA, the median LOS was 3 days, ranging from 3 days for workers with fractures, head injuries including skull fractures, crushing injuries, and all other injuries to a high of 4 days among workers with burn injuries. The total number of days hospitalized for the 611 workers reported by employers to OSHA was 3424 days. Overall hospital stays ranged from a low of 1 night to a high of 202 nights. The range of days for hospitalizations not reported to OSHA was 93 and the range for hospitalizations reported by employers to OSHA was 201 (data not shown). We used the Mann–Whitney *U* test to compare the distribution of the LOS (days) in the MMSIISS by whether the employer reported the injury to OSHA or not. Hospitalizations that were reported by employers to OSHA had a significantly longer distribution of lengths of stay across all injury/illness types including total injuries, except fractures (Table 3).

**Table 3:** Length of stay (LOS) by employer reporting status to OSHA of 2238 work‐related hospitalizations in the Michigan Multi‐Source Injury and Illness Surveillance System (MMSIISS), 2016–2018

	MMSIISS (*n* = 2238)					
	Did the employer report to OSHA?					
	Yes (*n* = 611)		No (*n* = 1627)		All (*n* = 2238)	
	Median and LOS, days					
Injury/illness type	Median	Total	Median	Total	Median	Total
Fracture	3	1331	3	2923	3	4254
Head injury and skull fracture	3*	523	2	1341	2	1864
Crushing	3*	482	2	741	3	1223
Burns	4*	467	3	584	3	1051
All other	3*	621	2	1422	3	2043
Total	3*	3424	3	7011	3	10,435

Abbreviation: OSHA, Occupational Safety and Health Administration.

* *p* < 0.01 based on the comparison of the distribution of the LOS by whether the employer reported to OSHA or not.

Employers in manufacturing (64.7%), wholesale trade (57.5%), and public administration (66.2%) were significantly more likely to report injuries and illnesses to OSHA compared to the overall compliance rate (45.1%), and employers in agriculture, forestry, fishing, and hunting (8.2%); construction (38.2%); finance and insurance (21.4%); real estate and rental and leasing (27.3%); administrative and support and waste management and remediation services (33.9%); arts, entertainment, and recreation (10.8%); accommodation and food services (20.8%) and all other services except public administration (20.3%) were significantly less likely to report injuries and illnesses to OSHA compared to the overall compliance rate (Table 4). The percentage of employers in compliance with the reporting requirement varied from a low of 8.2% in agriculture, forestry, fishing, and hunting to a high of 66.2% in public administration.

**Table 4:** Industry of 2887 work‐related hospitalizations by reporting source, Michigan 2016–2018

	Reporting source				
	MMSIISS only (*n* = 1627)	MMSIISS and employer reports to OSHA (*n* = 611)	Employer reports to OSHA only (*n* = 649)	All (*n* = 2887)	% Employers in compliance with reporting rule
Industry (NAICS)	No. (%)	No. (%)	No. (%)	No. (%)	% (95 CI)
Agriculture, forestry, fishing, and hunting (11)	112 (7.4)	5 (0.8)	5 (0.8)	122 (4.4)	▼8.2 (3.3–13.1)
Mining, quarrying, and oil and gas exploration (21)	8 (0.5)	2 (0.3)	3 (0.5)	13 (0.5)	38.5 (12–64.9)
Utilities (22)	12 (0.8)	6 (1.0)	13 (2.0)	31 (1.1)	61.3 (44.1–78.4)
Construction (23)	348 (22.8)	116 (19.0)	99 (15.4)	563 (20.3)	▼38.2 (34.2–42.2)
Manufacturing (31, 32, 33)	250 (16.4)	240 (39.4)	219 (34.1)	709 (25.5)	▲64.7 (61.2–68.2)
Wholesale trade (42)	54 (3.5)	37 (6.1)	36 (5.6)	127 (4.6)	▲57.5 (48.9–66.1)
Retail trade (44, 45)	126 (8.3)	50 (8.2)	41 (6.4)	217 (7.8)	41.9 (35.4–48.5)
Transportation and warehousing (48, 49)	81 (5.3)	31 (5.1)	38 (5.9)	150 (5.4)	46.0 (38–54)
Information (51)	10 (0.7)	3 (0.5)	3 (0.5)	16 (0.6)	37.5 (13.8–61.2)
Finance and insurance (52)	11 (0.7)	2 (0.3)	1 (0.2)	14 (0.5)	▼21.4 (−0.1 to 42.9)
Real estate and rental and leasing (53)	24 (1.6)	7 (1.1)	2 (0.3)	33 (1.2)	▼27.3 (12.1–42.5)
Professional, scientific, and technical services (54)	30 (2.0)	7 (1.1)	10 (1.6)	47 (1.7)	36.2 (22.4–49.9)
Management of companies and enterprises (55)	2 (0.1)	0	2 (0.3)	4 (0.1)	50.0 (1–99)
Administrative and support and waste management and remediation services (56)	113 (7.4)	26 (4.3)	32 (5.0)	171 (6.2)	▼33.9 (26.8–41)
Educational services (61)	49 (3.2)	15 (2.5)	24 (3.7)	88 (3.2)	44.3 (33.9–54.7)
Health care and social assistance (62)	77 (5.1)	11 (1.8)	40 (6.2)	128 (4.6)	39.8 (31.4–48.3)
Arts, entertainment, and recreation (71)	33 (2.2)	3 (0.5)	1 (0.2)	37 (1.3)	▼10.8 (0.8–20.8)
Accommodation and food services (72)	84 (5.5)	10 (1.6)	12 (1.9)	106 (3.8)	▼20.8 (13–28.5)
Other services (except public administration) (81)	55 (3.6)	7 (1.1)	7 (1.1)	69 (2.5)	▼20.3 (10.8–29.8)
Public administration (92)	44 (2.9)	31 (5.1)	55 (8.6)	130 (4.7)	▲66.2 (58–74.3)
Totals and overall compliance	1523	609	643	2774	45.1 (43.3–46.8)
Unknown	104	2	6	113	

*Note*: ▲ indicates significantly higher compliance rate than overall compliance rate and ▼ indicates significantly lower compliance rate than overall compliance rate.

Abbreviations: CI, confidence interval; MMSIISS, Michigan Multi‐Source Injury and Illness Surveillance System; NAICS, North American Industry Classification System; OSHA, Occupational Safety and Health Administration.

Large companies were significantly more likely to report the hospitalization to OSHA (68.4%), while small companies were significantly less likely to report the hospitalization to OSHA (32.9%) (Table 5).

**Table 5:** Company size of 2887 work‐related hospitalizations by reporting source, Michigan 2016–2018

	Reporting source				
	MMSIISS only (*n* = 1627)	MMSIISS and employer reports to OSHA (*n* = 611)	Employer reports to OSHA Only (*n* = 649)	All (*n* = 2887)	% Employers in compliance with reporting rule
Company size	No. (%)	No. (%)	No. (%)	No. (%)	% (95 CI)
Large (250+ employees)	212 (18.5)	196 (34.6)	262 (42.0)	670 (28.6)	▲68.4 (64.8–71.8)
Medium (11–249 employees)	660 (57.4)	310 (54.8)	286 (45.8)	1256 (53.7)	47.5 (44.7–50.2)
Small (1–10 employees)	277 (24.1)	60 (10.6)	76 (12.2)	413 (17.7)	▼32.9 (28.4–37.5)
Totals and overall compliance	1149	566	624	2339	50.9 (48.9–52.8)
Unknown	478	45	25	548	

*Note*: ▲ indicates significantly higher compliance rate than overall compliance rate and ▼ indicates significantly lower compliance rate than overall compliance rate.

Abbreviations: CI, confidence interval; MMSIISS, Michigan Multi‐Source Injury and Illness Surveillance System; OSHA, Occupational Safety and Health Administration.

**Figure 2:**
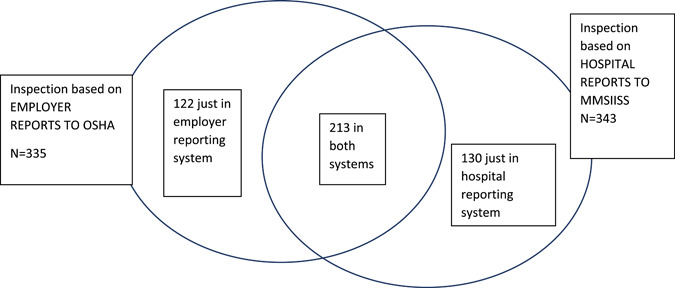
Overlap of 465 OSHA inspections based on workers in two reporting systems: Employer reports to OSHA and hospital reports to the Michigan Multi‐Source Injury and Illness Surveillance System (MMSIISS), Michigan 2016–2018. OSHA, Occupational Safety and Health Administration. [Color figure can be viewed at wileyonlinelibrary.com]

Four hundred and sixty‐five of the companies were inspected by MIOSHA where an inpatient hospitalization occurred; for 213 inspections the patient was reported both to the MMSIISS and by employers to OSHA, for 130 inspections the employer had not reported the hospitalization to OSHA, and for 122 inspections the MMSIISS did not have a record of a patient associated with that company (Table 6). Figure 2 shows the overlap of inspections across the two reporting systems. Three hundred and forty‐two (77.6%) of the companies inspected had fewer than 250 employees, with 91 (20.6%) having 1–10 employees, and 251 (56.9%) having 11–249 employees. There were 24 companies that had an unknown number of employees.

**Table 6:** Inspections, violations, and monetary penalty of 465 enforcement inspections of work‐related hospitalizations by reporting source and industry, and distribution by industry of companies in the MMSIISS that were not inspected, Michigan 2016–2018

	Reporting source						Companies in MMSIISS that were not inspected (*n* = 1895)^a^		
	MMSIISS only	MMSIISS and employer reports to OSHA	Employer reports to OSHA only	All inspections			Did the employer report to OSHA?		
	Inspections (*n* = 130)	Inspections (*n* = 213)	Inspections (*n* = 122)	Inspections (*n* = 465)	Total violations	Total penalty $	Yes (*n* = 398)	No (*n* = 1497)	All (*n* = 1895)
Industry (NAICS)	No. (%)	No. (%)	No. (%)	No. (%)			No. (%)	No. (%)	No. (%)
Agriculture, forestry, fishing, and hunting (11)	5 (3.8)	3 (1.4)	2 (1.6)	10 (2.2)	12	12,270	2 (0.5)	107 (7.7)	109 (6.1)
Mining, quarrying, and oil and gas extraction (21)	2 (1.5)	1 (0.5)	10	3 (0.6)	2	3850	1 (0.3)	6 (0.4)	7 (0.4)
Utilities (22)	0	1 (0.5)	3 (2.5)	4 (0.9)	6	25,250	5 (1.3)	12 (0.9)	17 (1.0)
Construction (23)	40 (30.8)	46 (21.6)	9 (7.4)	95 (20.4)	179	159,575	70 (17.7)	308 (22.1)	378 (21.1)
Manufacturing (31, 32, 33)	34 (26.2)	104 (48.8)	69 (56.6)	207 (44.5)	244	545,625	136 (34.3)	216 (15.5)	352 (19.7)
Wholesale trade (42)	7 (5.4)	15 (7.08)	10 (8.2)	32 (6.9)	31	51,300	22 (5.6)	47 (3.4)	69 (3.9)
Retail Trade (44, 45)	7 (5.4)	10 (4.7)	6 (4.9)	23 (4.9)	26	48,875	40 (10.1)	119 (8.5)	159 (8.9)
Transportation and warehousing (48, 49)	7 (5.4)	10 (4.7)	2 (1.6)	19 (4.1)	13	26,390	21 (5.3)	74 (5.3)	95 (5.3)
Information (51)	0	0	1 (0.8)	1 (0.2)	3	6750	2 (0.8)	11 (0.8)	13 (0.7)
Finance and insurance (52)	1 (0.8)	0	0	1 (0.2)	1	2250	2 (0.5)	10 (0.7)	12 (0.7)
Real estate and rental and leasing (53)	1 (0.8)	1 (0.5)	0	2 (0.4)	1	750	6 (1.5)	23 (1.7)	29 (1.6)
Professional, scientific, and technical services (54)	2 (1.5)	4 (1.9)	1 (0.8)	7 (1.5)	9	7700	3 (0.8)	28 (2.0)	31 (1.7)
Management of companies and enterprises (55)	0	0	0	0	0	0	0	2 (0.1)	2 (0.1)
Administrative and support and waste management and remediation services (56)	5 (3.8)	7 (3.3)	6 (4.9)	18 (3.9)	32	51,550	19 (4.8)	108 (7.8)	127 (7.1)
Educational services (61)	1 (0.8)	2 (0.9)	1 (0.8)	4 (0.9)	4	7950	13 (3.3)	48 (3.4)	61 (3.4)
Health care and social assistance (62)	5 (3.8)	1 (0.5)	5 (4.1)	11 (2.4)	8	14,400	10 (2.5)	72 (5.2)	82 (4.6)
Arts, entertainment, and recreation (71)	1 (0.8)	1 (0.5)	0	2 (0.4)	4	5750	2 (0.5)	32 (2.3)	34 (4.6)
Accommodation and food services (72)	4 (3.1)	3 (1.4)	1 (0.8)	8 (1.7)	7	14,350	7 (1.8)	80 (5.7)	87 (4.9)
Other services (except public administration) (81)	6 (4.6)	2 (0.9)	2 (1.6)	10 (2.2)	19	16,400	5 (1.3)	49 (3.5)	54 (3.0)
Public administration (92)	2 (1.5)	2 (0.9)	4 (3.3)	8 (1.7)	7	16,850	29 (7.3)	42 (3.0)	71 (4.0)
Total	130	213	122	465	608	1,017,835	396	1393	1789

Abbreviations: MMSIISS, Michigan Multi‐Source Injury and Illness Surveillance System; OSHA, Occupational Safety and Health Administration.

^a^ There were 106 individuals in the MMSIISS for which NAICS was unknown: two of whom were reported by their employer to OSHA and 104 of whom were not reported by their employer to OSHA.

Manufacturing (207, 44.5%) and construction (95, 20.4%) had the greatest number of inspections, followed by wholesale trade (32, 6.9%), retail trade (23, 4.9%), transportation and warehousing (19, 4.1%), administrative and support and waste management and remediation services (18, 3.9%), healthcare and social assistance (11, 2.4%), other services except for public administration (10, 2.2%), agriculture, forestry, fishing, and hunting (10, 2.2%), accommodation and food services (8, 1.7%), public administration (8, 1.7%), professional, scientific, and technical services (7, 1.5%), utilities (4, 0.9%), educational services (4, 0.9%), mining, quarrying, and oil and gas extraction (3, 0.6%), real estate and rental and leasing (2, 0.4%), arts, entertainment, and recreation (2, 0.4%), information (1, 0.2%), and finance and insurance (1, 0.2%). There was no union representing the workers at 360 (77.4%) of the companies inspected (data not shown). One hundred and twelve (24.1%) of the companies were cited for failure to report the hospitalization (data not shown). Companies were cited for 608 violations, 383 of which were serious violations. A total of $1,017,835 in penalty fines were paid, with an average of $2189. Of the 465 inspections, 246 (52.9%) employers had not abated the hazard directly associated with the injury requiring hospitalization in the months between the injury and the inspection, 49 (10.5%) employers were only cited for not reporting the hospitalization, 66 (14.2%) were only cited for violations not directly related to the hospitalization, and 104 (22.4%) employers were not issued any citations (Table 7). There were nine inspections associated with employer reports to OSHA that were cited for a violation of the reporting rule. In those cases, the employer reported the injury to the OSHA reporting system after the inspection began. Eight of the inspections were initiated due to a referral from an entity other than the employer and one company was a program‐related inspection.

**Table 7:** Enforcement inspections for 465 work‐related hospitalizations by reporting source and violation status, Michigan 2016–2018

	Reporting source			
	MMSIISS Only	MMSIISS and employer report to OSHA	Employer reports to OSHA only	Total
Violation status	No. (%)	No. (%)	No. (%)	No. (%)
Cited: Hazard directly related and not abated at the time of inspection	66 (50.8)	117 (54.9)	63 (51.6)	246 (52.9)
Only cited for not reporting the injury	40 (30.8)	7 (3.3)	2 (1.6)	49 (10.5)
Only cited for hazard(s) unrelated to the injury	8 (6.2)	32 (15.0)	26 (21.3)	66 (14.2)
Not cited for any violations	16 (12.3)	57 (26.8)	31 (25.4)	104 (22.4)
Total inspected	130	213	122	465
Not inspected	1497	398	784	3144

Abbreviations: MMSIISS, Michigan Multi‐Source Injury and Illness Surveillance System; OSHA, Occupational Safety and Health Administration.

Five narratives of injuries requiring hospitalization where an enforcement inspection was conducted.

An employee of a construction company was engaged in roofing work at a residential building. He was on a steep roof when he fell 22 feet onto the concrete driveway. Emergency services were called, and the employee was hospitalized for a fractured leg. The company was inspected 181 days after the hospitalization; they were issued a serious citation directly related to the injury for failure to provide guardrail systems with toe boards, safety net systems, or personal fall arrest systems when working on a steep roof. This hazard was still present at the time of inspection. This injury was identified only through the MMSIISS and had not been reported by the employer to OSHA. The company was cited for not reporting.

An employee was unloading a highway truck at the loading dock using a motorized hand truck. As the employee began to exit the highway truck, the truck pulled away from the loading dock. The employee and the hand truck fell out of the back of the truck onto the pavement. The employee was hospitalized with an orbital skull fracture as well as lacerations to the face and back of the head. The company was inspected 154 days after the hospitalization; they were issued a serious citation directly related to the injury for failure to ensure that a highway truck is not boarded before its brakes are set and no less than two wheels are blocked or restrained by other mechanical means to prevent the truck from movement. This hazard was still present at the time of inspection. This injury was identified only through the MMSIISS and had not been reported by the employer to OSHA.

An employee was assisting a coworker with moving a granite slab that weighed approximately 1000 pounds from a delivery truck to an outside storage bin. The employee was struck by the slab when it tipped forward from the forklift tines and fell on him. The employee was hospitalized for a collapsed lung and shoulder and rib fractures. The company was inspected 83 days after the hospitalization; they were issued a serious citation directly related to the injury for failure to lift or transport only a load that cannot fall out of a basket or container during the normal movements of the truck. This hazard was still present at the time of inspection. The company was also cited for failure to provide refresher training to an operator. This injury was identified only through the MMSIISS and had not been reported by the employer to OSHA. The company was cited for not reporting.

An employee was cutting branches off a tree. One of the branches that he was cutting fell on top of the employee, pinning him to the ground. The employee was hospitalized for multiple injuries. The company was inspected 115 days after the hospitalization and issued a serious citation directly related to the injury for failing to ensure the climbing employee remained tied in until the work is completed, and he returned to the ground. This hazard was still present at the time of inspection. This injury was identified only through the MMSIISS and had not been reported by the employer to OSHA.

An employee was engaged in a roofing activity. The employee unhooked his harness, lost his balance and fell 25 feet to the ground below. The employee was hospitalized for a fractured pelvis and tailbone. The company was inspected 65 days after the hospitalization and issued a serious citation for failure to provide a training program for each employee who might be exposed to a fall hazard. The company was given 30 days to correct the hazard. The company also was cited for four other‐than‐serious violations, including failure to have an accident prevention program, and failure to record recordable injuries and illnesses on the OSHA 300 injury and illness log. This injury was identified only through the MMSIISS and had not been reported by the employer to OSHA. The company was cited for not reporting.

## 4 CONCLUSION

Employer compliance with the reporting regulation could be examined because of the MMSIISS, which is a unique surveillance tool based on the requirement in Michigan that hospitals report work‐related injuries and illnesses. We used the MMSIISS data to understand the characteristics of the workers who were hospitalized for acute work‐related injuries and illnesses and to evaluate employer compliance with the reporting requirement of these injuries and illnesses. We are not aware of any similar evaluation for work‐related hospitalizations by OSHA on a national level or at the state level by other state‐plan states. A comparison of the employer reports to OSHA with the MMSIISS revealed poor compliance with the reporting requirement by employers. Employers did not report 1627 (56.4%) of the 2887 work‐related injury and illness hospitalizations from 2016 to 2018. Although 100% of hospitals in Michigan complied with the 1978 and 2010 Michigan regulations for reporting work‐related conditions, matching the MMSIISS data with the employer reports to OSHA (Figure 1) shows that there were 649 valid reports of hospitalizations from employers to OSHA that were not identified in the MMSIISS. For the 649 valid reports to OSHA that were not reported by hospitals to the MMSIISS, where the injury was a fracture, head trauma, or acute illness such as heat stroke, it appeared that the hospital failed to report either because the insurance of the injury or illness was not coded as workers' compensation or because the hospital did not correctly identify and report all the diagnostic codes requested. To definitively understand these 649 reports by employers to OSHA that were not found in MMSIISS would necessitate that OSHA require employers who report cases include the name of the hospital where the injured worker was admitted so their medical records could be reviewed.

While there has been no published evaluation of the new federal employer‐reporting rule for hospitalizations specifically, there are prior studies that have identified undercounting in employer‐based systems, the BLS SOII.^3^, ^4^, ^6^ Despite requirements for reporting, barriers to employer reporting have been identified, including employers not considering the injury work‐related, employee failure to report their injury to their employer due to fear of losing their job or other negative consequences, cost‐shifting injury costs to regular health insurance to avoid increased workers' compensation premiums and employer unawareness of reporting rules.^6^, ^7^ We would consider the circumstances very limited for when an employer would be unaware of an acute work‐related traumatic injury that caused a hospitalization and accordingly would ascribe the marked underreporting to lack of awareness, oversight, or deliberate disregard of the reporting rule. It is also possible that employers had actually reported the incident to workers' compensation or had logged the incident on the company's in‐house OSHA Injury and Illness 300 log and had mistakenly thought they were in compliance with the reporting requirement.

If one relied solely on the employer‐reported hospitalizations to OSHA to evaluate severe work‐related injuries and illnesses, a different picture of the characteristics, magnitude, and burden of the injuries and industries associated with these hospitalizations would be seen. For example, there was a significantly lower compliance rate with employers reporting to OSHA of head injuries including skull fractures compared to the total compliance rate (Table 2). Furthermore, the total LOS among the 611 hospitalizations reported by employers to OSHA amounted to only 3424 days, 32.8% of the 10,435 days among all the hospitalizations identified through the MMSIISS (Table 3). In addition, a significantly higher percentage of employers in manufacturing (64.7%), wholesale trade (57.5%), and public administration (66.2%) complied with the reporting requirement. Employers had a significantly lower compliance rate in: agriculture, forestry, fishing, and hunting (8.2%); construction (38.2%); finance and insurance (21.4%); real estate and rental and leasing (27.3%); administrative and support and waste management and remediation services (33.9%); arts, entertainment, and recreation (10.8%); accommodation and food services (20.8%); and other services except public administration (20.3%) (Table 4).

Work‐related hospitalizations are costly. Data from the Kaiser Family Foundation estimates that the average inpatient hospital stay per day in Michigan was $2245 for 2016, $2318 for 2017, and $2400 for 2018 (source: kff.org, accessed January 20, 2022). If we apply these average daily costs for each year to the number and LOS for individuals with work‐related hospitalizations, the estimated hospital costs for the 2238 workers in the MMSIISS would be $24,222,334; compared to $7,952,552 for the 611 workers reported by employers to OSHA, a threefold difference. Further, there are the hidden social and economic costs that are not quantified that are placed on workers who sustain these severe injuries and illnesses which result in hospitalization.^2^ The five narratives of workers with injuries resulting in hospitalization give some examples of the type and seriousness of these injuries.

The new reporting requirement was useful in identifying workers who sustained severe injuries and illnesses, which led to remediation in their workplaces when inspections were conducted that identified unsafe conditions. Follow‐up onsite workplace inspections benefited all workers at the companies where the workers were injured since the company was required to address all safety or health violations that were identified by the enforcement inspector. Even though inspections generally occurred 3 months after the injury, at the time of inspection, 52.9% of the companies inspected had not abated the hazard that was directly related to the injury (Table 7). The companies inspected were required to provide proof that the 608 violation items were corrected, thereby leading to a safer workplace. Better compliance with reporting by employers^8^ and more complete reporting from hospitals would result in improved working conditions for all employees at the companies where workers sustain severe injuries.

There are a few potential limitations. It is possible that some of the work‐related hospitalizations in the MMSIISS did not meet the OSHA requirement for reporting. However, the medical records generally provided detailed narratives about the injuries, including in some cases pictures of the injuries, that provided sufficient information to determine that the hospitalizations met the OSHA reporting requirement. In accordance with the employer reporting regulation, we excluded any hospitalizations that involved motor vehicle collisions, the self‐employed or involved nonacute conditions such as musculoskeletal conditions. Workers' compensation paid for 58.7% of the 2238 hospitalizations in the MMSIISS; for the ones where workers' compensation was not the payer, we identified an employer, confirmed the person was not self‐employed, and the medical record clearly indicated the injury occurred at work. Letters and a questionnaire were sent to hospitalized individuals to complete when the medical record did not list the employer's name or type of industry, or we wanted to clarify whether an OSHA inspection should be conducted. Letters were sent to 415 individuals; all 74 who completed the questionnaire reported their injury was acute, work‐related, and they had reported the hospitalization to their employer.

This is the first evaluation of the federal OSHA severe injury and illness reporting requirement for hospitalizations. Because there is a comprehensive system of reporting in place by hospitals in Michigan, we were able to assess employer compliance with the reporting regulation for hospitalizations. We have identified a gap of approximately 50% in reporting by employers to OSHA. Given the usefulness of these reports to identify workplace hazards, it would be beneficial to increase employer compliance with the reporting requirement at a national level. There is no reason to expect that employers in other states are more or less likely to comply with the regulation. Given the usefulness of inspections initiated because of work‐related hospitalization reporting, efforts to increase compliance should include both increased enforcement of the regulation and educational efforts to increase awareness among employers of the reporting rule. It would be beneficial for OSHA to add the hospital to which the worker was admitted to their reporting system to ensure more complete reporting when cross‐referencing hospital‐reported and employer‐reported workers. Additional resources would be required for both the increased outreach efforts and enforcement inspections that would subsequently be conducted.

The low compliance with the regulation was across all 3 years, with no appreciable improvement in compliance from 2016 to 2018. Disproportionate reporting in manufacturing, wholesale trade, and public administration as well as companies with a large number of employees suggests employer lack of awareness of the requirement among employers less familiar with workplace safety and health, or who do not have a dedicated health and safety specialist. Better reporting lays the groundwork to fully characterize the industries where injuries and acute illnesses result in hospitalization, which can then be used to target and evaluate preventive interventions in the workplace.

## CONFLICTS OF INTEREST

The authors declare that there are no conflicts of interest.

## DISCLOSURE BY AJIM EDITOR OF RECORD

John D. Meyer declares that he has no conflicts of interest in the review and publication decision for this article.

## AUTHOR CONTRIBUTIONS

Ms. Mary Jo Reilly compiled the data and wrote the first draft of the manuscript. Dr. Kenneth D. Rosenman provided the concept idea for the manuscript and contributed substantially to the writing and edits. Dr. Ling Wang was responsible for the statistical analyses as well as edits to the manuscript. All three authors contributed substantially to the revised manuscript.

## ETHICS APPROVAL AND INFORMED CONSENT

This public health activity was considered exempt by the Michigan State University Human Subjects Board.

## DISCLAIMER

The contents of the manuscript do not necessarily reflect the views of the funding agency.

## Data Availability

Research data are not shared.
